# The Protective Influence of Bilingualism on the Recovery of Phonological Input Processing in Aphasia After Stroke

**DOI:** 10.3389/fpsyg.2020.553970

**Published:** 2021-01-05

**Authors:** Miet De Letter, Elissa-Marie Cocquyt, Oona Cromheecke, Yana Criel, Elien De Cock, Veerle De Herdt, Arnaud Szmalec, Wouter Duyck

**Affiliations:** ^1^Department of Rehabilitation Sciences, Ghent University, Ghent, Belgium; ^2^Department of Neurology, Ghent University Hospital, Ghent, Belgium; ^3^Psychological Sciences Research Institute, Université catholique de Louvain, Louvain-la-Neuve, Belgium; ^4^Department of Experimental Psychology, Ghent University, Ghent, Belgium

**Keywords:** bilingualism, phonology, aphasia, recovery, neuroplasticity

## Abstract

Language-related potentials are increasingly used to objectify (mal)adaptive neuroplasticity in stroke-related aphasia recovery. Using preattentive [mismatch negativity (MMN)] and attentive (P300) phonologically related paradigms, neuroplasticity in sensory memory and cognitive functioning underlying phonological processing can be investigated. In aphasic patients, MMN amplitudes are generally reduced for speech sounds with a topographic source distribution in the right hemisphere. For P300 amplitudes and latencies, both normal and abnormal results have been reported. The current study investigates the preattentive and attentive phonological discrimination ability in 17 aphasic patients (6 monolinguals and 11 bilinguals, aged 41–71 years) at two timepoints during aphasia recovery. Between the two timepoints, a significant improvement of behavioral language performance in both languages is observed in all patients with the MMN latency at timepoint 1 as a predictive factor for aphasia recovery. In contrast to monolinguals, bilingual aphasic patients have a higher probability to improve their processing speed during rehabilitation, resulting in a shortening of the MMN latency over time, which sometimes progresses toward the normative values.

## Introduction

Recovery from stroke-related aphasia follows arbitrarily described stages, starting with the acute and subacute phase (<6 months poststroke) and slowly progressing to the chronic phase (≥6 months poststroke). In the acute and subacute stages, language improvement is related to a combination of spontaneous recovery and speech therapy, whereas the chronic recovery stage is mainly determined by the effects of speech therapy. Lesion location and size, aphasia type and severity, and to some extent the nature of early hemodynamic response are the most important factors that determine recovery, whereas the predictive values of gender, age, handedness, and education are a matter of debate (Watila and Balarabe, 2015).

Phonological comprehension displays the most strong and consistent recovery between 5 and 9 months poststroke (Robson et al., 2019). Consequently, aphasics with significant impairments in auditory and phonological abilities in subacute and early chronic stages may be at risk of poor language comprehension outcomes (Robson et al., 2019).

Acoustic–phonological processing consists of two processes: acoustic (prephonological) processing and phonological input processing. Acoustic (prephonological) processing is related to activity in the left midposterior superior temporal gyrus (STG) and sulcus (STS), and their underlying white matter, and is functionally associated with relatively simple acoustic structures such as modulated tones, frequency sweeps, and harmonic stimuli (Hall et al., 2000; Menon et al., 2002; Husain et al., 2004), as well as with the analysis of auditory–phonological information (Binder et al., 2000; Benson et al., 2001). Phonological input processing consists of phonological analysis and discrimination and phonological short-term memory (Robson et al., 2011). Although individual differences have been reported, the underlying network for auditory discrimination on word level is situated in the posterior STG (Boatman and Miglioretti, 2005) and the left and right supramarginal gyri (Hartwigsen et al., 2010). Phonological short-term memory supports a wide range of linguistic behaviors including the maintenance of information during sentence and discourse processing (Martin and White, 2005). The network supporting auditory phonological short-term memory also involves the supramarginal gyri and the posterior STG (Paulesu et al., 1993; Henson et al., 2000; Leff et al., 2009). In addition, two prefrontal areas are involved in phonological short-term memory, namely, the inferior frontal gyrus and the supplementary motor area (Brendel et al., 2010; Peeva et al., 2010). Although both structures are motor planning areas, they are activated during purely receptive phonological working memory tasks as well (Rauschecker et al., 2008; Strand et al., 2008).

As the acoustic–phonological processing possesses limited capacity for neuroplasticity, residual functional integrity after stroke is an important prognostic indicator for comprehension recovery (Robson et al., 2019). Recently, a longitudinal prospective study explored the cognitive dynamics underpinning changes in comprehension from the subacute to the chronic stage. Interestingly, the authors found that speech comprehension recovery (in Wernicke’s Aphasia) results from reorganization of the remaining language comprehension network rather than from partial recovery of language- or cognitive-specific domains (Robson et al., 2019).

Temporal aspects of reorganization of language- or cognitive-specific domains after stroke can be measured by means of event-related potentials (ERPs). Two of the most frequently used ERPs in cognitive and linguistic neuroscience are the preattentive (unconscious) mismatch negativity (MMN) and the attentive (conscious) P300. The MMN is a negative deflection in the auditory ERP at about 150–200 ms, whereas the P300 refers to a positive deflection in the auditory ERP at around 300 ms. Both can be elicited by stimuli that are “deviant” in an oddball task, which makes these approaches especially suited to study verbal sound discrimination.

Aerts et al. (2013) used these brain potentials in healthy volunteers to compare three phonemic contrasts that can be identified in Dutch consonants: (1) place of articulation (PoA), (2) manner of articulation, and (3) phonation of the consonants (voicing). The authors concluded that during auditory phoneme discrimination, all three phonemic contrasts are differently affected by the influence of age, with PoA being the most resistant and voicing being the most vulnerable.

Mismatch negativity and P300 of the PoA may detect amplitude or latency deviations even when behavioral ceiling effects have been reached using pen-and-paper tasks, which means that these two potentials are highly sensitive to subtle phonological deficits (Aerts et al., 2015). This is in line with the subjective experience of aphasic patients who indicate that they do not feel fully recovered, although behavioral assessments fail to show remaining deficits (Aerts et al., 2013). Functional correlates of amplitude attenuation and delayed latency time are interpreted as a functional reduction (Csépe et al., 2001; Ilvonen et al., 2004; Dejanović et al., 2015) and a delayed onset (Luck, 2014) of linguistic and cognitive processes, respectively.

In addition to the amplitude and latency of an ERP, the cerebral topography of the elicited activity may support the formulation or modification of therapy guidelines. In 20 patients with chronic aphasia, phonological training correlated with an increased synaptic gain in the left STG, whereas patients with more severe speech comprehension disturbances showed strengthening of bidirectional connections between the left and right STG (Woodhead et al., 2017).

Aphasia occurs almost as frequently in multilingual as in monolingual individuals (Alladi et al., 2016). In contrast to monolingual aphasic patients, the diagnostic procedure in bilingual aphasics depends on the proficiency of the native language (L1) and the other acquired languages (L2, L3, …). Early or simultaneous bilinguals have a “native (L1) and native-like (L2)” language (Li and Moyer, 2008). Other multilinguals acquire their native and second language consecutively, which is most common in European countries. They are typically called late bilinguals.

It has been shown that language switching and language control, implied by bilingualism, recruit the same neural architecture as non-verbal, higher-order cognitive control mechanisms (Abutalebi and Green, 2008; Declerck and Philipp, 2015; for review see Calabria et al., 2019). As a consequence, a recent line of research has investigated whether bilingualism may have beneficial consequences for cognition, beyond the linguistic domain, i.e., the so-called bilingual advantage (e.g., de Bruin et al., 2015). And, indeed, it has been shown that multilingualism may offer protection against cognitive decline, as shown by later clinical manifestation of neurodegenerative diseases such as Alzheimer dementia (Woumans et al., 2015). This protection is also referred to as “cognitive reserve.” Cognitive reserve has also been described as a “protective” factor in aphasia patients, as multilingual aphasics were shown to have a better recovery after stroke (Alladi et al., 2016).

Recovery of multilingual aphasic patients is a challenging topic because brain lesions do not necessarily affect L1 and L2 in the same way (Verreyt et al., 2013; Van der Linden et al., 2018a,b), and the recovery pattern for each language is unpredictable as it depends on multiple influencing factors, such as the age of language acquisition, frequency of language exposure, linguistic similarity between one’s languages, premorbid proficiency, and even education level (for review, see Kuzmina et al., 2019). In contrast to Hillis and Tippett (2014) and Plowman et al. (2012); Watila and Balarabe (2015) suggest that there is no clear and consistent relationship between education level and aphasia severity or recovery. However, in patients with severe aphasia, Marinelli et al. (2017) distinguished three different cognitive profiles. They found that the group with a significantly higher education level showed higher percentages of accuracy for all cognitive functions, predicting a better and faster recovery of linguistic abilities.

There is also strong evidence for an important role of premorbid language proficiency in bilinguals (Kuzmina et al., 2019). Stronger connections between language and cognitive control networks were found for the language showing a better recovery. Unfortunately, ERP studies focusing on the neuroplasticity of language and language control networks during aphasia recovery after stroke in bilinguals are still lacking.

In conclusion, monolingual and bilingual aphasia patients seem to have a different neuroplastic capacity during language recovery after stroke, which is probably related to a different stimulation of their language control network during language development and use (Abutalebi and Green, 2008). Aphasia recovery depends on multiple influencing factors that need to be identified. Neuroplasticity in language-related networks can be measured during the different stages of recovery with language-related brain potentials. The current study aims to investigate the impact of bilingualism on the recovery of phonological input processing after stroke, measured behaviorally and with ERPs (preattentive and attentive oddball paradigms). A correlation between the electrophysiological and behavioral assessment of language performance in the recovery stage will be investigated, as well as the question whether premorbid factors correlate with the phonologically related potentials (MMN and P300).

We hypothesize that bilingual aphasic patients will rely on cognitive control systems, which results in a faster phonological processing than monolinguals. This assumption could lead bilinguals to demonstrate higher ERP amplitudes and latencies that are closer to normative values in comparison to monolingual aphasia patients.

## Materials and Methods

In order to approach the above mentioned research questions, a number of sub-questions were formulated:

1 Does bilingualism have an impact on the cerebral reorganization of phonological input processing after stroke?
1.1 Is there a correlation between the values obtained at T1 and T2 for MMN and P300 in aphasic patients as a group?
1.2 Is there a significant difference between monolinguals (*n* = 6) and bilinguals (*n* = 11) in the evolution of ERP parameters between T1 and T2 (MMN and P300 amplitude and latency) in aphasic patients as a group?
1.3 How do monolingual and bilingual aphasia patients differ from normative values for phonological input measures during aphasic recovery?
1.4 Do factors such as age, education level, multilingualism, L2 proficiency, and language use correlate with the ERP parameters (MMN and P300)?
2. Is there a correlation between the electrophysiological and behavioral results?

### Patients

Seventeen right-handed aphasic patients (13 male, 4 female, average age 56 ± 15 years) with aphasia resulting from their first-ever stroke participated in the study. The mean time between stroke onset and test moment 1 (T1) was 37 months. The background information for the patients in the recovery stage is included in Supplementary Appendix 1. No other neurological or psychiatric disorders than a first stroke were reported. The group consists of 6 monolingual and 11 bilingual non–native-like aphasia patients. All types of aphasia were included here, provided that the functional comprehension was intact. Neither cognitive deficits other than the language disturbances nor hearing problems that could interfere with the auditory investigations were mentioned. In addition, the patients were evaluated for their ability to auditorily discriminate between consonants/b/and/g/, which was necessary to perform the ERP language paradigm for this study.

The patients were evaluated in the chronic phase poststroke (>6 months after onset). They were tested behaviorally and electrophysiologically at two time points, with 5 months in between. Between T1 and test moment 2 (T2), some but not all patients received aphasia therapy with a frequency of one to five sessions of 60 min a week during a maximum of 2 years as defined by the reimbursement restrictions in Belgium.

Ethical approval was obtained from the Ghent University Hospital and conducted according to the latest version of the Declaration of Helsinki. Written consent from the patient or a legal representative was given if comprehension difficulties were too severe.

### Procedure

Data of aphasia patients 1–9 were collected prospectively, whereas data of patients 10–17 years were studied retrospectively. The language proficiency questionnaire was administered retrospectively for all patients, provided they were able to understand the questionnaire (four patients were not able to understand the questionnaire, see Supplementary Appendix 2). The patients judged their L2 proficiency before and after (T2) stroke on a self-rating scale from 0 (no proficiency) to 10 (maximal proficiency) on the following language tasks: spontaneous speech, reading, writing, and comprehension. As both groups were studied in separate settings with different diagnostic pen-and-paper testing (see section “Behavioral Analysis”), they only had the electrophysiological registration procedure in common.

#### Electrophysiological Registration, Tasks, and Analysis

Dutch-based ERP paradigms were presented to the monolingual and bilingual aphasic groups because language perception in a second language was assumed to be deeply affected by the phonemic structure of the native language (Moreno et al., 2008), and the normative data for phonological input processing were available only for the native language of the aphasia groups (Dutch) (Aerts et al., 2013).

Electroencephalogram data were recorded using the BrainVision recorder software (Brain Products, Germany) and an Easycap including 32 Ag/AgCl-electrodes, namely, Fp1/2, Fpz, F3/4, F7/8, Fz, FC1/2, FC5/6, C3/4, T7/8, Cz, CP1/2, CP5/6, P3/4, P7/8, Pz, TP9/10, POz, O1/2, and Oz. All electrodes were placed on the scalp according to the international 10–20 system. The online reference was FCz, and the ground electrode was AFz. Impedances were kept below 10 kΩ. We used an actiCHamp amplifier (Brain Products, Germany), and data were digitized at 500 Hz.

During registration, two phonological oddball tasks were presented containing the standard stimulus/b/and the deviant stimulus/g/(differing in terms of the phonemic contrast PoA). Both phoneme discrimination tasks have been developed by our research group (Aerts et al., 2013). In order to elicit MMN, phoneme discrimination was evaluated without attention of the patients, who had to watch a silent movie and ignore the auditory stimuli. For the P300, patients had to push a button whenever they heard a deviant stimulus. The sequence of tasks was counterbalanced across the patients. In both tasks, the standard and deviant stimulus probability was 0.80 and 0.20, respectively, relative to 750 (MMN) and 150 trials (P300). All stimuli (recorded by a female native speaker of Dutch) had a duration of 250 ms and were binaurally presented at a listening level of 70 dB with Apple Inc., earphones. Stimuli were randomly presented and two or more deviants could never succeed each other. An interstimulus interval (ISI) of 500 ms was used in the MMN task, and an ISI of 2000 ms was selected in the P300 task.

Analyses of MMN and P300 were performed using BrainVision Analyzer 2.1 (Brain Products, Munich, Germany). First, a high-pass filter of 0.5 Hz (slope 12 dB/octave), a low-pass filter of 30 Hz (slope 48 dB/octave), and a notch filter of 50 Hz were applied. Artifacts caused by eye blinks and horizontal eye movements were identified and removed by means of independent component analysis. Data were re-referenced offline to the mean of the left and right mastoids. Next, a separate analysis for standard and deviant stimuli was performed from segmentation. The epochs were 500 and 1100 ms long for MMN and P300 task, respectively. All epochs consisted of a 100-ms prestimulus baseline period, used for baseline correction. All data exceeding ±100 μV were semiautomatically rejected. Finally, an average of standard trials and of deviant trials was created. For the inattentive phoneme discrimination task (MMN), further analysis was performed on difference waves (difference between the standard trials and the deviant trials). For the attentive phoneme discrimination task, further analysis was performed on the average of deviant trials. Peak latencies and amplitudes were calculated semiautomatically in a component-specific time window, namely, 100–300 ms for MMN and 300–700 ms for P300. Moreover, we focused on electrode positions for which normative data in healthy individuals (Aerts et al., 2013) are available (MMN: Cz and Fz; P300: Pz) in order to compare the electrophysiological results of the patients.

#### Behavioral Analysis

Data about the L2 proficiency are included in Supplementary Appendix 2. The prospectively studied group (patients 1–9) was evaluated with the Dutch version of the Comprehension Aphasia Test (CAT-nl; Swinburn et al., 2005). The retrospectively studied aphasia group (patients 10–17) was studied with the Dutch version of the Aachen aphasia test (AAT; Graetz et al., 1993) supplemented with the auditory discrimination and short-term memory tasks of the Dutch version of the Psycholinguistic Assessments of Language Processing in Aphasia (Kay et al., 1992). Behavioral assessment results are included in Supplementary Appendices 3, 4.

#### Statistics

Statistical analysis was performed in SPSS version 25. As patients were studied in separate settings with different aphasia batteries, no statistical analysis could be made on the level of behavioral (pen-and-paper testing) data.

In order to detect whether monolinguals and bilinguals deviate from normative data on the level of MMN and P300 paradigms (Aerts et al., 2013), a Fisher exact test was used (comparison of two categorical variables with *n* < 40 and not all obtained frequencies >5). Depending on the results of the distribution, a paired Student’s *t*-test or Mann–Whitney *U* test was used in order to compare ERP parameters within the whole group of monolingual and bilingual aphasic patients. To compare the subgroups of 11 multilinguals and 6 monolinguals, a Mann–Whitney *U* test was applied. In order to check a potential correlation between the subscores on the CAT-Nl/AAT and the ERP parameters, Spearman correlation test was used.

In order to detect if influencing factors such as age, education level, multilingualism, L2 proficiency, language use, and CAT-Nl/AAT subscores have a predictive value for the outcome of the ERP parameters (MMN and P300), a single linear regression analysis was performed for every factor consecutively (*p* < 0.2). This first selection allowed performing a multiple linear regression analysis in an attempt to obtain a predictive regression line.

## Results

1. Does bilingualism have an impact on the cerebral reorganization of phonological input processing after stroke?
  1.1 Is there a correlation between the values obtained at T1 and T2 for MMN and P300 values in the aphasic patients as a group?

**FIGURE 1:**
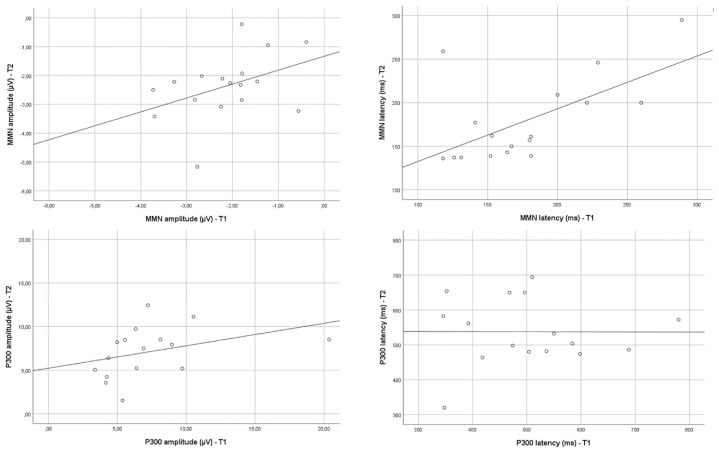
Scatterplots of “the amplitude/latency – MMN/P300 at T1” versus “amplitude/latency – MMN/P300 at T2.”

*Results*: Between T1 and T2, a positive linear correlation could be detected for MMN_latency time (*r* = 0.58; *p* = 0.015) and P300_amplitude (*r* = 0.61; *p* = 0.01). A weak positive linear correlation was also found for MMN_amplitude between T1 and T2 (*r* = 0.41; *p* = 0.099). No correlation was found for P300 latency values between T1 and T2 (*r* = −0.04; *p* = 0.988). Scatterplots are graphically presented in Figure 1.

1.2 Is there a significant difference between monolinguals (*n* = 6) and bilinguals (*n* = 11) in the evolution of ERP parameters between T1 and T2 (MMN and P300 amplitude and latency)?

*Results*: Latency MMN paradigm: a significant difference could be observed for the MMN latency between monolinguals [med(IQR) = 11.00 (−3.50 to 88.50)] and bilinguals [med(IQR) = −18.50 (−22.50 to 9.00), *U* = 11.50; *p* = 0.048]. Monolinguals showed an increase of latency time at T2 relative to T1, whereas bilinguals showed a decrease in latency time at T2.

1.3 Do monolingual and bilingual aphasic patients differ from normative values for phonological input during aphasia recovery?

*Results*: The number of monolinguals performing within normative values decreases for most parameters (MMN latency time, P300 amplitude, and latency time) at T2, whereas the number of bilinguals situating within normative values is stable or increases in T2 (Table 1).

**TABLE 1:** Number of mono- and bilinguals for which ERP parameters are situated within the normative values on T1 and T2.

		Monolinguals (*n* = 6)		Bilinguals (*n* = 11)	
			
Test moment		T1	T2	T1	T2
MMN	Amplitude	2	4	10	9
	Latency time	4	3	9	10
P300	Amplitude	2	1	7	9
	Latency time	4	2	7	9

1.4 Do factors such as age, education level, multilingualism, L2 proficiency, and language use correlate with the ERP parameters (MMN and P300)?

Results: In order to correlate premorbid L2 proficiency with electrophysiological parameters (Kuzmina et al., 2019), the aphasia patients had to go back up to 7 years (5–89 months) to retrospectively judge their premorbid L2 proficiency.

We assume that this long interval makes the judgments unreliable. Therefore, we decided to exclude the premorbid L2 status of the analyses and to focus only to the data poststroke at T2.

The latency time of the MMN at T1 (*p* = 0.015) and T2 (*p* = 0.018) was found to be a predictive factor for the parameter “proficiency L2 at T2” (=proficiency_post).

2 Is there a correlation between the electrophysiological and behavioral results?

As ERP paradigms correlate with auditory word and sentence comprehension, we are mainly interested in correlations of the ERP values with these subtests.

2.1 Is there a correlation on the level of aphasia severity between the monolingual and bilingual group?

Results: No significant difference (*p* = 0.359) for aphasia severity (token test) between the monolingual and bilingual group at T1 could be demonstrated.

2.2 Is there a correlation between electrophysiological results and those of pen-and-paper tasks?

*Results* (pen-and-paper tasks: patient 1–9: CAT-NL; patient 10–17: AAT): In patients 1–9, a positive correlation (*r* = 0.557) was found between P300 latency time and auditory word comprehension (CAT-NL) at T1. In patients 10–17, a positive correlation could be detected between aphasia severity (AAT-token test) and MMN latency on T1 (*r* = 0.739).

## Discussion

This study reports on behavioral and electrophysiological assessments of phonological input processing in 17 aphasia patients (11 bilinguals and 6 monolinguals) in the aphasia recovery stage. In summary, the results of the study demonstrate that MMN latency time can be considered as the most reliable and predictive ERP parameter for aphasia recovery. The MMN latency time correlates with aphasia severity (the shorter the latency time, the milder the aphasia) and act as the best predictor for aphasia outcome and L2 proficiency (the shorter the latency time, the better the outcome). In contrast to monolinguals, bilingual aphasia patients have a higher probability to improve their processing speed during rehabilitation, resulting in a shortening of MMN latency over time, which sometimes progresses toward normative values. These results partially confirm our hypotheses. We cannot confirm the hypothesis of a possible premorbid predictive role of L2 during aphasia recovery (Kuzmina et al., 2019) as the aphasia patients had to go back up to 7 years to retrospectively judge their premorbid L2 proficiency. We assume that this timeframe makes the judgments unreliable.

From an electrophysiological perspective, there was a positive linear correlation for MMN latency time and P300 amplitude between the values at T1 and T2 across all aphasic patients as a group. At T2, the phonologically elicited potentials in monolinguals were characterized by an increase and in bilinguals by a decrease of MMN latency time, suggesting that the monolingual group needed more time to perform the task at T2, whereas the bilingual group needed less time than at T1. The positive impact of bilingualism on phonological input processing after stroke could be explained by the fact that cognitive–linguistic interconnections give overlapping support to multiple languages (Alladi et al., 2016; Paplikar et al., 2018). While bilingual aphasic patients rely on higher-order cognitive control systems to restore their linguistic networks, monolingual aphasics can only address the intact parts of the linguistic network, resulting in increased MMN latency and less recruitment of neurons underlying linguistic networks.

The presence of auditory discrimination deficits in some patients in T1 could be related to an involvement of the left temporal cortex in the lesion localization of all these patients. Comparing behavioral and electrophysiological results in patient 1–9, a positive correlation (*r* = 0.557) was found between P300 latency time and auditory word comprehension (CAT-NL) at T1, suggesting better results for aphasic patients at T1 when they had more time to process the auditorily presented word.

The question remains whether this comprehension impairment is related to the type and severity of aphasia. Regarding aphasia taxonomy (aphasia with comprehension problems/intact comprehension), five of the nine patients (in patients 1–9) presented without phonological comprehension problems (Broca and Anomic) versus four of nine with phonological comprehension problems (Wernicke and global aphasia). The delayed auditory discrimination processing in patients with as well as without comprehension problems suggests that not the comprehension problem, but rather the underlying cognitive problems influence the results on comprehension performance in this group. In patients 10–17, a positive correlation could be detected between the aphasia severity, which is related to comprehension (AAT-token test), and the MMN latency on T1 (*r* = 0.739), confirming our hypothesis that the patients with severe aphasia have disturbed underlying cognitive networks (Marinelli et al., 2017) inhibiting the speed processing in auditory discrimination task. Moreover, bilingual aphasia patients consequently rely on higher-order cognitive control systems to restore their linguistic networks, resulting in a short MMN latency time on T2, whereas monolinguals do not.

In bilinguals, higher-order cognitive control is required to select, maintain, and switch between the two languages. Preservation of the cognitive control system poststroke may have a favorable influence on L2 proficiency. As our patients have non–native-like higher L2 proficiency, our results could therefore be interpreted as a manifestation of cognitive reserve (Hillis and Tippett, 2014; Watila and Balarabe, 2015), suggesting a higher accuracy for all cognitive functions and faster recovery of linguistic abilities (Marinelli et al., 2017). To our knowledge, this is the first time that the relationship between a higher L2 proficiency poststroke and linguistic-related potentials during aphasia recovery (MMN latency) was demonstrated. Further research on the relationship between linguistic-related potentials and influencing variables such as socioeconomic status, general intelligence, literacy level, cultural influences, age, and gender is still needed in larger and more balanced groups.

This kind of longitudinal research is challenging because of many issues, such as the recruitment of a sufficiently large group of monolingual and bilingual aphasia patients who are equally balanced for age, gender, education, language proficiency, etc. Not all patients perform adequately on the behavioral and electrophysiological testing, and some have to be excluded during the procedure because of medical or personal reasons. Therefore, we preferred to limit the analysis to group analyses across recovery stages.

In the current study, results of the monolingual aphasic group have to be interpreted with caution as this group is rather small (*n* = 6). For the European countries, it will be a challenge to obtain a balanced cohort with mono-and bilingual aphasic patients. Moreover, although aphasia has been reported to be almost as frequent in multilinguals as in monolinguals, at least for the Indian population (Alladi et al., 2016), the population in the Western European countries seems to evolve from predominantly monolingual to predominantly bilingual aphasic patients over the last two decades. The most obvious reasons of increasing bilingualism in the elderly population and in aphasia patients are the increasing level of multilingual education in school that starts already at a primary school level and the increased number of patients with an immigration background and naturally developing bilingualism resulting from globalized multimedia exposure, mostly in English.

Concerning the usefulness of ERP in clinical settings, ERP responses could become an interesting tool for the follow-up of neuroplasticity in L1 and L2 during language recovery. As multilingual aphasic patients activate their language control networks, adequate cognitive ERP paradigms must be developed and included in the clinical evaluation of multilingual patients. Therefore, we suggest developing a validated, integrated cognitive–linguistic ERP battery for multilingual aphasia diagnostics. In addition, language specific cognitive–linguistic ERP batteries would allow developing and/or fine-tuning cognitive–linguistic therapy guidelines for aphasia rehabilitation.

## Conclusion

This study reports on a behavioral and electrophysiological follow-up of language in monolingual and bilingual aphasic patients in the recovery stage after stroke. The results of this study suggest that MMN latency can be considered as an independent predictor of L2 proficiency during aphasia recovery. The advantage of L2 proficiency for aphasia recovery after stroke paves the way to follow-up research on cognitive training of healthy elderly people.

## Data Availability Statement

The original contributions presented in the study are included in the article/Supplementary Material, further inquiries can be directed to the corresponding author/s.

## Ethics Statement

The study was approved by the Ethical Committee of the Ghent University Hospital. The patients/participants provided their written informed consent to participate in this study.

## Author Contributions

MD: writing of consecutive drafts. E-MC: data analysis. OC: first draft. YC: statistical analysis. ED and VD: data acquisition. AS: draft revision. WD: draft revision. All authors contributed to the article and approved the submitted version.

## Conflict of Interest

The authors declare that the research was conducted in the absence of any commercial or financial relationships that could be construed as a potential conflict of interest.
