# Supplementary material for: The Protective Influence of Bilingualism on the Recovery of Phonological Input Processing in Aphasia After Stroke
Source: Front Psychol. 2021 Jan 5;11:553970. doi: 10.3389/fpsyg.2020.553970 (PMC7814870; doi:10.3389/fpsyg.2020.553970)
Supplement: Supplementary file 1 [file Data_Sheet_1.docx]

**Appendix 1. Background information for the patients**

Table 1. Background information and assessment results for the patients

| Patient | Age  T1 | Sex | Handed-ness | Type of aphasia  on T1 | T1 post stroke onset (months) | Lesion localization | Stroke type | Education | L1 | L2 |
| --- | --- | --- | --- | --- | --- | --- | --- | --- | --- | --- |
| 1 | 43 | male | Right | Wernicke | 8 | Left FP + I | ischemic | HE | Dutch | English |
| 2 | 41 | male | Right | Wernicke | 11 | Left T + I | ischemic | SS | Dutch | English |
| 3 | 49 | male | Right | Amnestic | 53 | Left lentic-stri | hemorrhagic | HE | Dutch | French |
| 4 | 67 | male | Right | Broca | 85 | Left FP | hemorrhagic | SS | Dutch | English |
| 5 | 60 | male | Right | Broca | 74 | Left FTP | ischemic | HE | Dutch | French |
| 6 | 51 | male | Right | Global | 89 | Left PT + I+ TO | ischemic | SS | Dutch | - |
| 7 | 52 | male | Right | Broca | 65 | Left MCA | hemorrhagic | SS | Dutch | English |
| 8 | 67 | male | Right | Global | 79 | Left FT | ischemic | HE | Dutch | Spanish |
| 9 | 61 | female | Right | Global | 5 | Left FP + I | ischemic | SS | Dutch | - |
| 10 | 62 | male | Right | Amnestic | 41 | Left PT | ischemic | HE | Dutch | French |
| 11 | 71 | male | Right | Wernicke | 20 | Left T | hemorrhagic | HE | Dutch | English |
| 12 | 46 | male | Right | Wernicke | 33 | Left P + I + lentic-stri | ischemic | HE | Dutch | English |
| 13 | 55 | male | Right | Wernicke | 30 | Left T | hemorrhagic | HE | German | French |
| 14 | 63 | male | Right | Wernicke | 5 | Left PT | ischemic | SS | Dutch | - |
| 15 | 46 | female | Right | Wernicke | 18 | Left MCA + lentic-stri | ischemic | HE | Dutch | - |
| 16 | 71 | female | Right | Global | 6 | Left MCA + lentic-stri + I | hemorrhagic | HE | Dutch | - |
| 17 | 51 | female | Right | Global | 18 | Left MCA + lentic-stri | ischemic | SS | Dutch | - |

FP=frontoparietal; I=Insular; T=temporal; Lentic-stri= lenticulo-striatal; FTP=frontotemporoparietal; PT=parietotemporal; TO=temporo-occipital; MCA=middle cerebral arteria; FT=frontotemporal; HE=higher education; SS=secondary school; T1= test moment 1

Appendix 2. Self-rating L2 proficiency before and after (T2) stroke

| Patient | L2 proficiency before stroke (/10) | | | | L2 proficiency at T2 (/10) | | | |
| --- | --- | --- | --- | --- | --- | --- | --- | --- |
|  | Speech | Reading | Writing | Compre-hension | Speech | Reading | Writing | Compre-hension |
| 1 | 10 | 6 | 8 | 2 | 8 | 1 | 8 | 8 |
| 2 | 8 | 4 | 8 | 3 | 8 | 0 | 8 | 8 |
| 3 | 7 | 1 | 8 | 1 | 8 | 3 | 8 | 5 |
| 4 | 7 | 7 | 7 | 7 | 7 | 7 | 7 | 7 |
| 5 | 8 | 2 | 8 | 2 | 8 | 0 | 8 | 5 |
| 6 | / | / | / | / | / | / | / | / |
| 7 | 7 | 2 | 6 | 0 | 4 | 0 | 6 | 5 |
| 8 | 10 | 0 | 10 | 0 | 10 | 0 | 10 | 0 |
| 9 | 10 | 6 | 8 | 2 | 8 | 1 | 8 | 8 |
| 10 | 7 | 4 | 8 | 7 | 7 | 5 | 7 | 5 |
| 11 | 6 | 2 | 6 | 3 | 6 | 3 | 7 | 6 |
| 12 | 9 | 5 | 9 | 7 | 9 | 4 | 9 | 7 |
| 13 | 10 | 4 | 10 | 5 | 10 | 1 | 10 | 6 |
| 14 | / | / | / | / | / | / | / | / |
| 15 | 8 | 3 | 8 | 3 | 7 | 1 | 7 | 2 |
| 16 | / | / | / | / | / | / | / | / |
| 17 | / | / | / | / | / | / | / | / |

Legend: Scale from 0 to 10, with 0= no proficiency and 10= maximal proficiency; L2= second language; T2 = test moment 2 (5 months between T1 and T2)

Appendix 3. Behavioral assessment results at T1 and T2 (CAT-NL) in patients 1-9.

| Pt | CAT-NL results at T1 | | | | | CAT-NL results at T2 | | | | | |
| --- | --- | --- | --- | --- | --- | --- | --- | --- | --- | --- | --- |
|  | Token Test  /50 (pc) | Repeti-tion  total  /61 | Writing  Total  /82 | Naming  total  /58 | Comprehension (aud total)  /66 | Token Test  /50 (pc) | Repeti-tion  total  /61 | Writing  Total  /82 | Naming  Total  /58 | Comprehension  (aud total)  /66 |  |
| 1 | 41 (32) | 25 | 54 | 42 | 56 | 37 (41) | 26 | 57 | 48 | 59 |  |
| 2 | 47 (16) | 46 | 56 | 35 | 33 | 42 (30) | 36 | 47 | 33 | 43 |  |
| 3 | 11 (84) | 57 | 58 | 48 | 64 | 15 (77) | 60 | 60 | 51 | 61 |  |
| 4 | 5 (93) | 53 | 62 | 54 | 65 | 7 (90) | 55 | 60 | 54 | 63 |  |
| 5 | 12 (83) | 50 | 59 | 52 | 62 | 7 (90) | 54 | 62 | 52 | 61 |  |
| 6 | 44 (24) | 15 | 54 | 3 | 51 | 44 (24) | 21 | 53 | 11 | 62 |  |
| 7 | 6 (91) | 40 | 59 | 50 | 66 | 10 (85) | 49 | 59 | 53 | 62 |  |
| 8 | 40 (35) | 29 | 49 | 8 | 48 | 28 (59) | 32 | 58 | 6 | 49 |  |
| 9 | 33 (50) | 43 | 56 | 45 | 59 | 25 (63) | 40 | 57 | 48 | 61 |  |

Appendix 4. Behavioral assessment results at T1 and T2 (Aachen Aphasia Test) in patients 10-17.

| Pt | AAT-results at T1 | | | | | AAT-results at T2 | | | | | |
| --- | --- | --- | --- | --- | --- | --- | --- | --- | --- | --- | --- |
|  | Token Test  /50 (pc) | Repeti-tion  /150 (pc) | Reading & writing /90 (pc) | Naming  /120 (pc) | Compre-hension  120 (pc) | Token Test | Repeti-tion  /150 (pc) | Reading & writing /90 (pc) | Naming  /120 (pc) | Compre-hension  120 (pc) |  |
| 10 | 23 (65) | 75 (25) | 64 (63) | 82 (55) | 91 (64) | 14 (79) | 134 (80) | 88 (97) | 108 (92) | 114 (98) |  |
| 11 | 41 (32) | 52 (15) | 58 (57) | 93 (70) | 87 (55) | 7 (90) | 136 (83) | 87 (95) | 119 (100) | 119 (100) |  |
| 12 | 43 (27) | 75 (25) | 64 (63) | 82 (55) | 91 (64) | 22 (67) | 139 (85) | 90 (100) | 112 (98) | 115 (99) |  |
| 13 | 48 (2) | 30 (10) | 35 (36) | 0 (4) | 33 (3) | 41 (32) | 87 (34) | 49 (49) | 39 (4) | 59 (17) |  |
| 14 | 46 (18) | 115 (58) | 71 (70 | 92 (69) | 83 (48) | 5 (88) | 148 (98) | 90 (100) | 114 (99) | 116 (99) |  |
| 15 | 36 (43) | 127 (73) | 39 (41) | 99 (80) | 98 (77) | 36 (43) | 130 (75) | 43 (72) | 110 (96) | 102 (83) |  |
| 16 | 34 (48) | 75 (25) | 48 (48) | 28 (22) | 76 (37) | 26 (61) | 83 (30) | 60 (59) | 73 (46) | 92 (66) |  |
| 17 | 32 (51) | 0 (1) | 0 (3) | 0 (4) | 81 (45) | 26 (61) | 65 (19) | 33 (34) | 43 (28) | 86 (52) |  |

Legend: pt = patient; T1 = test moment 1, T2 = test moment 2 (5 months between T1 and T2)
